# Disease of the Antrum of Highmore

**Published:** 1851-07

**Authors:** R. D. Couch


### ARTICLE XV.

*Disease of the Antrum of Highmore.* By R. D. Couch, Esq., M. R. C. S. L.

I observed, at page 32 of the new and improved series of your journal, a very interesting report from King's College Hospital, of disease of the antrum. From Mr. Fergusson's remarks, the disease appears to be a rare one, and for this reason I have forwarded the subjoined case of a similar disease.

Mary P., a healthy country woman, aged 52, received a blow about three years ago on the right cheek, about one inch and a half below the eye. She suffered a great deal of pain for some weeks; this became much better in about six weeks, but never entirely subsided. At the end of six months the face began to swell, and then she felt occasional fits of severe pain in the bones of the face and along the alveolar edge. This continued for two years the swelling was not very large. About twelve months since, the pain and swelling greatly increased. At this time, it appears that she was repeatedly leeched and blistered, and, as these failed to afford her much relief, various other local applications were used, but without much benefit. A short time before I saw her, a tooth had been extracted, without any good effect. On examination, it appeared that the disease was situated in the antrum, and that the pain and swelling were most probably caused by a confined fluid within the cavity. I could not discover that any discharge had taken place on the extraction of the tooth, though it was a decayed one. A small trochar was passed through the socket of the extracted tooth, and then forced through the bony plate which separated it from the maxillary sinus. A discharge of sero-purulent matter followed, which was very offensive to the smell. After this, she continued comparatively easy for some months; but the disease not subsiding in the manner I anticipated, another examination was made, and over the socket of the extracted molar, there was a small abscess. On opening this, the bone was discovered to be diseased, through which there was a small opening. The gums were then divided from their attachments by a scalpel, and the whole of the diseased bone was then laid bare. The opening was enlarged with a bistoury, and afterwards with a bone forceps, and the cavity laid bare. There was some difficulty, though the mouth was a large one, in raising the lips sufficiently to get a clear view of the whole part. This, however, having been done, the cavity was exposed. The whole of the lining membrane appeared villous, and showed signs of long continued congestion and inflammation. The inferior portion was ulcerated, and a thick grumous fluid was oozing from the part. At the posterior portion a mass of flesh seemed to occupy the whole place. On exploring this part, the mass seemed pendulous, with a contracted peduncle. This was brought down with a torsion forceps, and removed with a bistoury as high up as the root. Having examined again the surrounding bone, and removed all that seemed diseased, the lip was brought down and the wound dressed. A fetid discharge frequently occurred through the nostril, and fluids at first would occasionally pass from the mouth through the sinus into the nose; but this gradually became less frequent, and the patient is now recovered.

In this case the disease seems to have arisen from a blow over the antrum, and the diseased tooth may, perhaps, have been the predisposing cause to the peculiar form which it assumed. In these days of chloroform, the operation described as being adopted by Mr. Fergusson may not be objected to; but it seems to be quite as severe as the importance of the case will allow; and, in the opinion of some, perhaps a little more so. The operation reported from King's College seems to be better fitted for a removal of some large portion of the superior maxilla than disease of the antrum alone; but, having chloroform at command, such views as these cannot have the weight they would have had in former years. I offer this case, however, only as an instance of a rare disease.—*Med. Times.*

